# Nonmechanical Infrared Beam Steering Using Blue Addressed Quantum Dot Doped Liquid Crystal Grating

**DOI:** 10.1186/s11671-016-1816-8

**Published:** 2017-01-13

**Authors:** Xiangru Wang, Xiaoping Huang, Ziqiang Huang, Liang Wu, Jiyang Shang, Qi Qiu, Shuanghong Wu

**Affiliations:** 1 School of Optoelectronic information, University of Electronic Science and Technology of China, Chengdu, 610054 China; 2 School of Physical Electronics, University of Electronic Science and Technology of China, Chengdu, 610054 China; 3 Research Institute Electronic Science and Technology, University of Electronic Science and Technology of China, Chengdu, 610054 China; 4 Shanghai Aerospace Electronic Technology Institute Shanghai, Shanghai, 201109 China

**Keywords:** Quantum dot, Liquid crystal grating, Nonmechanical beam steering

## Abstract

We present a scheme of nonmechanical laser beam steering using ZnS/InP quantum dots doping nematic liquid crystal as the optical recording film. Because of its internal electric field generated by blue laser-induced charge carrier distribution, liquid crystal molecules are reoriented to form a phase grating which make the incident angle steer to the angle as we desire. Being a nonmechanical programmable laser beam steering, the anisotropy of the relative permittivity tensor and blue laser-induced electric carriers play a significant effect in determining the reorientable liquid crystal molecule and reconfigurable phase modulation of the gratings, that determines the steering angle and steering efficiency.

## Background

Nonmechanical laser beam steering promises significant benefits to many free-space laser application, especially on those where precise pointing and tracking system are required, such as free-space optical (FSO) communication, Lidar [1]. Many nonmechanical steering techniques have been suggested and studied including liquid crystal phase grating [2], MEMS [3], nanoscale optical antenna [4], gratings [5], fiber array [6], and so on.

Meanwhile, phase grating using the material of liquid crystal (LC) technology has undergone tremendous development not only on a single programmable device [7–10] but also on some integrated photonic devices due to its excellent electro-optic properties [11]. To be one of the most promising candidates to be a nonmechanical steerer, narrow steering range has been still a crucial short legs on one device. To accurately and efficiently steer optical beams over a large field of regard (FOR), the scheme of cascade system was proposed from an idea of N-nary numerical cascade system having multiple devices (M). And this M-bit-N-nary system was suggested to be used as numerical domain selection, and the ordinary liquid crystal phase grating was used to be domain filling [12]

To realize a N-nary numerical laser steering, a couple of devices was invented including polarized prism [13], liquid crystal polarization grating (LCPG) (passive and active) [14, 15], multiplex volume Bragg grating (VBG) [16]. Generally, these numerical N-nary devices are all based on a general physics that: device should have a specific grating structure where the steering property is sensitive to the state of incident beam, and the state parameter could be polarization, incident angle, angular momentum, and so on. So, the value of N to realize a practical N-nary system is very limited to be binary for passive LCPG, quasi-ternary for active LCPG, and ternary for multiplex VBG. More importantly, steering angles of these N-nary devices are fixed and one-time written, but none is truly erasable or fully programmable.

**Fig. 1:**
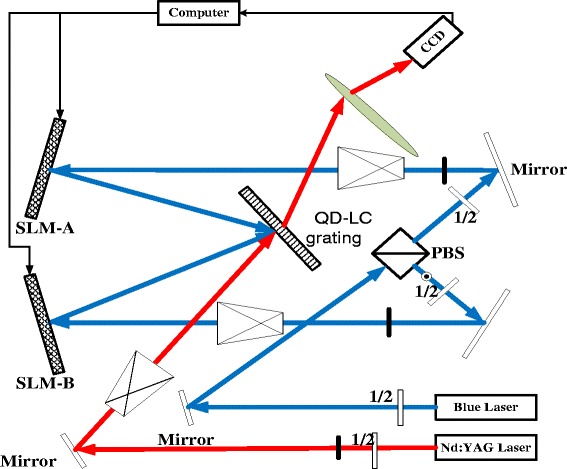
Optical setup of QD-LC laser steering

In this letter, quantum dot doped liquid crystal (QD-LC) as the rewritable recording medium by blue laser [17], a novel optical setup is proposed to succeed a programmable diffractive grating on this QD-LC layer using two spatial light modulators to steer the writing coherent blue beams. As shown in Fig.1, on this novel blue laser-addressed grating, not only the grating period is programmable but also grating direction. To utilize the first order grating, the diffraction efficiency is required on a very high level. Meanwhile, its response time is on the level of few milliseconds [18].

## Methods

**Fig. 2:**
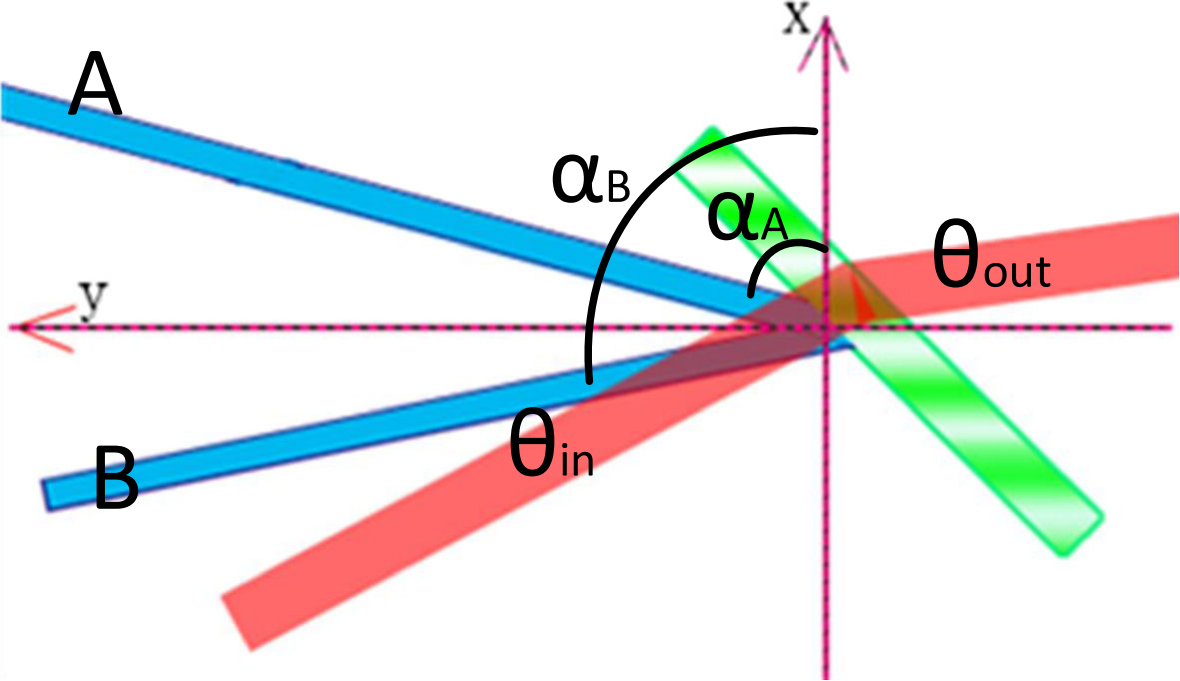
Coordinate and angles definition of QD-LC laser steering system

In our experimental setup shown in Fig. 1, from an optically pumped single frequency MSL-FN-473 solid laser from CNI company with a wavelength of *λ*_*B*_ = 473 nm and coherence length of longer than 50 m, the writing laser source are split into two beams which are set to be both s-polarized and intersected at an angle of *α*_*A*_ and *α*_*B*_ with respect to the *x*-axis, respectively, where the coordinate system is defined as shown in Fig. 2. The incident angle of these two writing beams are realized by its given spatial light modulators (SLM) SLM-A and SLM-B. The incident plane (x-o-y plane) defined by the wave vectors of the two recording beams is perpendicular to the cell substrate. When their corresponding SLM does not load any data, i.e., direct reflection, QD-LC sample is titled at 45 with respect to the bisector of the two recording beams so that there is a projection of the grating vector onto the electric field vector to separate the electron-hole pairs easily.

The interference region is read by a linearly polarized coherent beam with a wavelength of 1064 nm, which is from a single mode coupled Nd:YAG laser with a power of more than 10 mW. Meanwhile, the reading near infrared beam propagates through the phase modulated QD-LC device with the angle of *θ*_in_ with respect to the *x*-axis. After the phase modulation and fully interference, the reading beam is tilt to the direction of *θ*_out_.

**Fig. 3:**
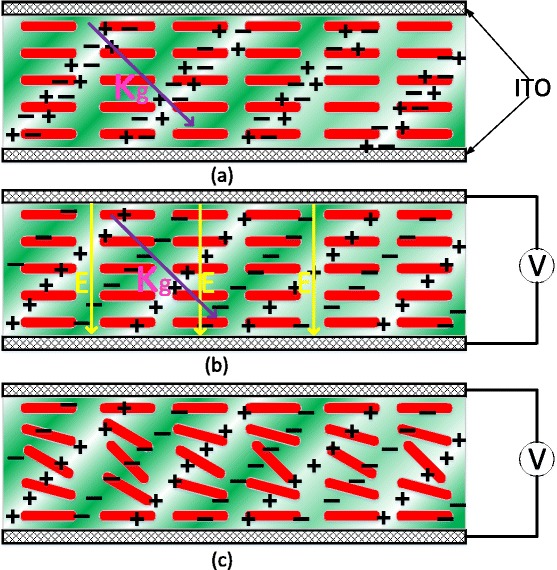
Photorefractive mechanism of QD-LC grating. **a** Hole-electron pairs generation. **b** Hole-electron pairs separation. **c** Liquid crystal reorientation

Being a rewritable holography recording medium, quantum doping liquid crystal is similar withrevisable optical writing media such as photochromic polymers [17]. Because of its fast response and high photoconductivity [18], it has already been applied on the field of holographic glass-free 3D display. The grating formation is induced by orientational photorefractive (PR) effect related to photoexcitation and transportation of charge carriers [19]. Charge carriers (holes and electrons) are generated by pumped blue photons so as to form an internal field in the QD-LC mixture and boundary layer interface. The distribution of charge carriers is modulated by interferometric fringe intensity, the grating direction of which is *κ*_*g*_, as shown in Fig. 3a. Driven by an external bias electric field *E*, the holes and electrons are trapped in the bright and dark regions, respectively, so as to form an enhanced spatially varying space charge field, as shown in Fig. 3b. Then, this spatially varying space charge field reorient the liquid crystal molecules to form a birefringence grating as show in Fig. 3c.

With the purpose of programming grating period and grating direction, according to the optical designed above, the value of grating direction *θ*_*g*_ and grating period *Λ* are determined by

1 $$ {\theta}_g=\frac{\alpha_B+{\alpha}_A}{2}\hbox{-} \frac{\pi }{2} $$

2 $$ \frac{\lambda {}_B}{2\varLambda }= \sin \left(\frac{\alpha_B-{\alpha}_A}{2}\right) $$

Meanwhile, according to the grating equation, we know, according to the theory of Kogelnik [20], if the incident beam propagates along the direction of first order diffraction angle

3 $$ {\theta}_{\mathrm{in}}=\frac{\alpha_B+{\alpha}_A}{2}+ \arcsin \frac{\lambda_{\mathrm{IR}}}{\varLambda } $$

it goes out with the angle perpendicular to the grating direction, being determined by

4 $$ {\theta}_{\mathrm{out}}={\theta}_g-\pi /2 $$

Substitute (1) and (3) into (4) and (2), respectively, Eqs. (2) and (4) are rewritten that:

5 $$ \sin \frac{\alpha_B-{\alpha}_A}{2}=\frac{\lambda_{\mathrm{B}}}{2{\lambda}_{\mathrm{IR}}} \sin \left({\theta}_{\mathrm{out}}-{\theta}_{\mathrm{in}}\right) $$

6 $$ {\alpha}_B+{\alpha}_A=2{\theta}_{\mathrm{out}}+2\pi $$

For the given *θ*_out_ we desire, *θ*_in_ is always unchanged after system initially being setup, the angles of two coherent writing beams *α*_*A*_ and *α*_*B*_ can be obtained according to Eqs. (5) and (6), and realized by the nonmechanically spatial light modulator, where the phase on each electrode is governed by the method of variable blazing grating [2]. With respect to the normal direction of their given spatial light modulator, the steering angles of A and B *α*_*A*/*B*_ and *α*_*B*_ are determined by

7 $$ \sin {\alpha}_{A/B}=\frac{\varDelta {\phi}_{A/B}}{k_0d} $$

where *Δϕ* is the phase step between two adjacent pixels, *d* is the pixels period, *k*_0_ is the vacuum wave number of addressing blue laser, i.e., the ideal phase retardation on the n-th electrode is the remainder of *nΔϕ* divided by 2π.

The blue addressed QD-LC phase plate is perfectly fabricated according the standard process of liquid crystal cell. Two ITO electrode coated fused silica were well pretreated after UV and DI water cleaning. After mechanical rubbing on the polyimide layer coated on ITO substrates for homogeneous alignment, these two substrates are well aligned and spaced with a thickness of 30 μm by spherical spacers. Then, the working medium QD-LC material is a mixture of nematic liquid crystal and ZnS/InP quantum dots, where the weight doping concentration is almost 1:20. Therein, the ZnS/InP quantum dots must be firstly pretreated in a vacuum drying oven for roughly 10 h at 70 ^o^C so as to evaporate the chloroform and solvent. Finally, this mixture of QD-LC was filled in the empty liquid crystal cell mentioned above via capillary action at room temperature. Stainless pins are usually banded on the edge of ITO substrates to connect with electric driving circuit or driver.

## Results and discussion

Initially, the incident angle of *α*_*A*_ and *α*_*B*_ are 85°and 95°(not loading data), respectively. Grating direction is *x* axis, i.e., *θ*_*g*_ = 0, According to Eqs. (2) and (3), the initial incident angle of infrared reading beam *θ*_in_ is 113.1°. After loading, the driving data generated from computer to SLM-A and SLM-B, the steering out angle is measured by CCD camera and easily triangle geometrics. Table 1 shows the experimental measured result of steering angle versus different steering angle of SLM-A and SLM-B. It shows a good agreement with the theoretical results as we designed.

**Table 1:** Steering angle versus incident angles of blue laser

Desired *θ*_out_	Theoretical results of		Meas. results of *θ*_out_
	*α*_*A*_	*α*_*B*_	
−85.0°	81.04°	98.96°	−84.99°
−87.5°	83.02°	96.98°	−87.50°
−90.0°	85.00°	95.00°	−90.01°
−92.5°	86.99°	93.01°	−92.49°
−95.0°	88.99°	91.01°	−95.01°

**Fig. 4:**
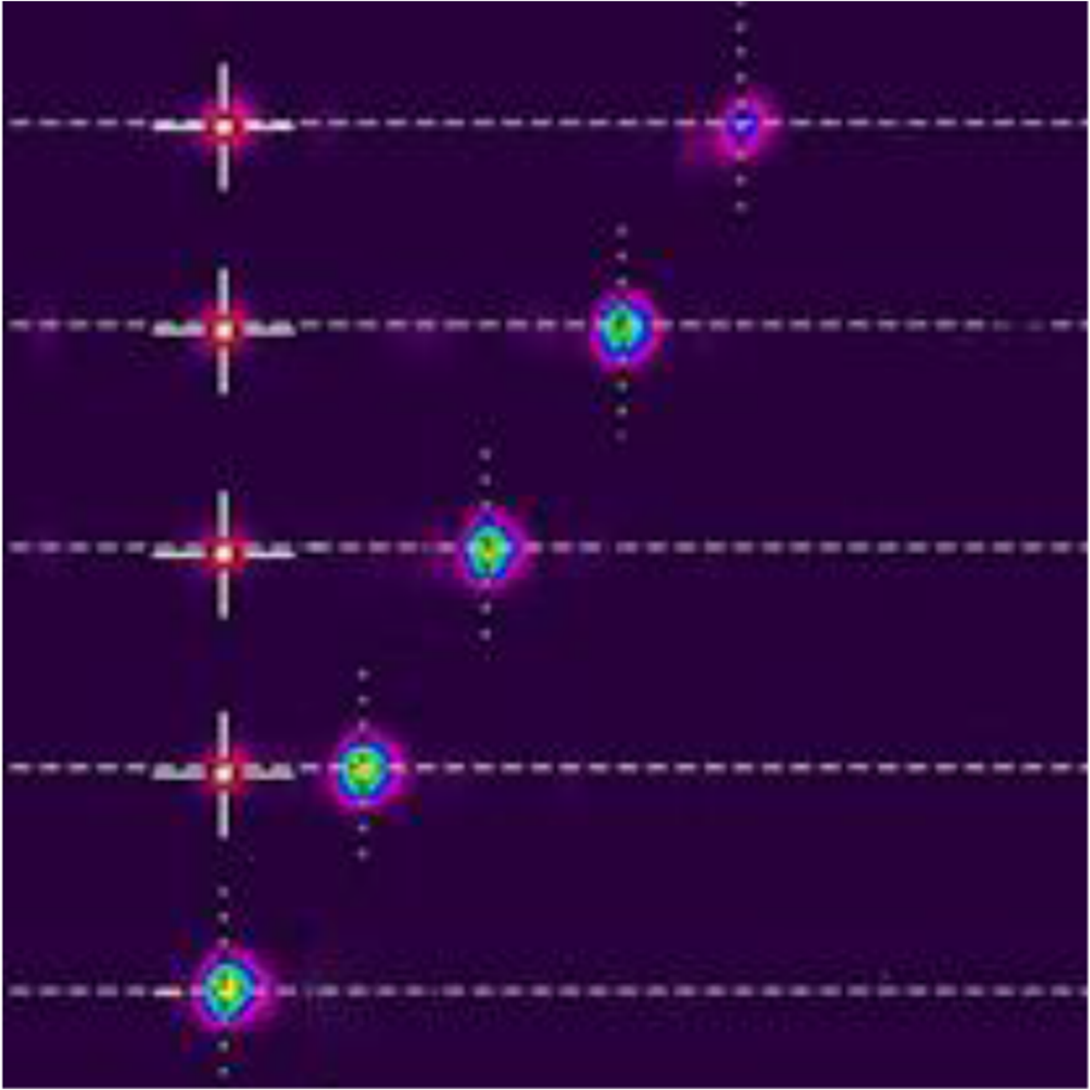
Captured far field of steering beam

Meanwhile, the measured results and theoretical results are plotted in Fig. 4. Meanwhile, Fig. 4 shows the measured steering angles versus desired angle which is linear increasing. Furthermore, the captured beam profiles of some issues are listed on the materials we used here are widely available, and the method is scalable, making this approach promising for future use in large size, dynamic, wide angle nonmechanical beam steering.

Importantly, because of the injection of blue addressing laser on the device, those functional layers would play significant roles on blue laser absorption where it could result into heat deposition, particularly on the ITO layer. The additional heat deposition may lower the threshold of working laser power. However, addressing blue laser is usually on the level of mill watt per square centimeter, comparing with few watts or even kilo watts of the working infrared lasers on the potential applied fields such as laser communication, lidar, laser weapon. On the heat deposition, the contribution of addressing laser is quite small so that it is a neglectable issue. Meanwhile, with an infrared camera, we have not found any significant temperature rising.

## Conclusions

In summary, we have established a study of nonmechanical laser beam steering system, where the phase grating of reading beam is recorded by using ZnS/InP quantum dots doping nematic liquid crystal film. We have shown that the birefringence of the liquid crystal is modulated by the internal electric field generated by the blue laser-induced charge carrier distribution. The behavior of reorientation of liquid crystal molecule forms a phase grating which makes the incident angle steer to the angle as we desire, so as to a nonmechanical laser beam steering. In particular, the anisotropy of the relative permittivity tensor and blue laser-induced electric carriers play a key role in determining the reorientable liquid crystal molecule and reconfigurable phase modulation of the gratings, so that it would influence the efficiency and steering range of nonmechanical laser beams steering.

## Abbreviations

FOR: Field of regard
FSO: Free-space optical
LC: Liquid crystal
LCPG: Liquid crystal polarization grating
MEMS: Micro electric mechanical system
QDLC: Quantum dot doped liquid crystal
SLM: Spatial light modulators
VBG: Volume Bragg grating
